# Recommendations for clinical decision-making when offering exoskeletons for community use in individuals with spinal cord injury

**DOI:** 10.3389/fresc.2024.1428708

**Published:** 2024-08-14

**Authors:** Derrick Onate, Cassandra Hogan, Kathryn Fitzgerald, Kevin T. White, Keith Tansey

**Affiliations:** ^1^ Lifeward Inc., Marlborough, MA, United States; ^2^ United States Department of Veterans Affairs, James A. Haley Veterans’ Hospital, Tampa, FL, United States; ^3^ Department of Neurology, University of South Florida, Tampa, FL, United States; ^4^ Center for Neuroscience and Neurological Recovery, Methodist Rehabilitation Center, Jackson, MS, United States; ^5^ United States Department of Veterans Affairs, G.V. (Sonny) Montgomery VA Medical Center, Jackson, MS, United States; ^6^ Department of Neurosurgery, University of Mississippi Medical Center, Jackson, MS, United States

**Keywords:** personal powered exoskeleton, mobility, community ambulation, quality of life, spinal cord injury

## Abstract

Approved in 2014 by the Food and Drug Administration (FDA) for use with a trained companion, personal powered exoskeletons (PPE) for individuals with spinal cord injury (SCI) provide an opportunity for the appropriate candidate to ambulate in their home and community. As an adjunct to wheeled mobility, PPE use allows those individuals who desire to ambulate the opportunity to experience the potential physiological and psychosocial benefits of assisted walking outside of a rehabilitation setting. There exists, however, a knowledge gap for clinicians regarding appropriate candidate selection for use, as well as who might benefit from ambulating with a PPE. The purpose of this paper is to provide guidance for clinicians working with individuals living with SCI by outlining an expert consensus for a PPE decision-making algorithm, as well as a discussion of potential physiological and psychosocial benefits from PPE use based on early evidence in publication.

## 1 Introduction

There are an estimated 291,000 people in the United States living with a spinal cord injury (SCI), with approximately 17,730 new cases recorded annually. SCI varies widely in severity, and approximately 32% of injuries are considered complete by clinical exam, with no motor or sensory function below the level of injury. The remaining 68% includes varying degrees of incomplete injuries, with some preservation of motor and/or sensory function below the level of injury (1). Prediction of functional ambulation after SCI is dependent on many factors and highly nuanced, but the ability to ambulate remains of high importance for many people living with SCI, regardless of injury level or severity (2).

Most individuals with a SCI that is complete by clinical exam, and many with incomplete injuries, reach a neurological recovery plateau at a motor capacity insufficient for unassisted standing or walking and require manual or powered wheelchairs to navigate their environments quickly with the least amount of energy expenditure. For patients who experience enough motor recovery to allow for some functional ambulation, there are several unpowered orthotics and bracing options available that allow for assisted ambulation, but often result in significant energy expenditure and lower gait speeds limiting their use primarily to household ambulation or static standing activities (3).

In 2014, the first robotic exoskeleton for home and community use was made commercially available. These wearable devices allow individuals to stand and walk with the assistance of a trained companion, providing complimentary mobility solutions for wheelchair patients who prioritize ambulation for their quality of life. While early evidence points to the potential physiological and psychosocial benefits of powered exoskeleton use, there exists little to no research on guidelines for introducing the use of powered exoskeletons to patients. While authors have presented the long-term goal of identifying guidelines and informing training procedures for powered exoskeleton prescription using an established RCT as a potential structure for future studies (4), the lack of currently available literature to help guide clinicians is a limitation that requires further research to develop evidence-based guidelines for both rehabilitation and personal use. The goal of this paper is to bridge this knowledge gap by providing expert consensus from clinicians experienced in personal powered exoskeleton (PPE) prescription. Two Doctors of Physical Therapy and their supervising physician from the James A Haley Veteran's Hospital, a Veterans Affairs (VA) SCI/D Center, which has a well-developed, dedicated Robotics and Advanced Technology program, as well as a physician neuroscientist at the G.V Montgomery VA Medical Center with extensive experience in gait robotics, were selected based on their experience and expertise in prescribing PPE within an established program. To help clinicians identify appropriate PPE patients, the aim of this paper is to outline a proposed decision-making process based on available literature and expert opinion, as well as discuss potential physiological and psychosocial benefits for patients. While there has not been sufficient time or device use to evaluate *post hoc* the success rate of the proposed algorithm, without guidance the field will have a hard time generating enough exoskeleton experiences to evaluate or for new prescribing algorithms to be proposed and tested against the one put forth here. It is the hope of the authors that this paper will provide guidance to increase the utilization of PPE, and in turn help develop better evidence-based tools and algorithms to guide use and prescription. While powered exoskeletons are also available for rehabilitation purposes, it is beyond the scope of this paper to discuss their utilization as a locomotor training tool for individuals working toward neurologic and functional recovery.

## 2 Algorithm for a PPE trial

**Figure 1:**
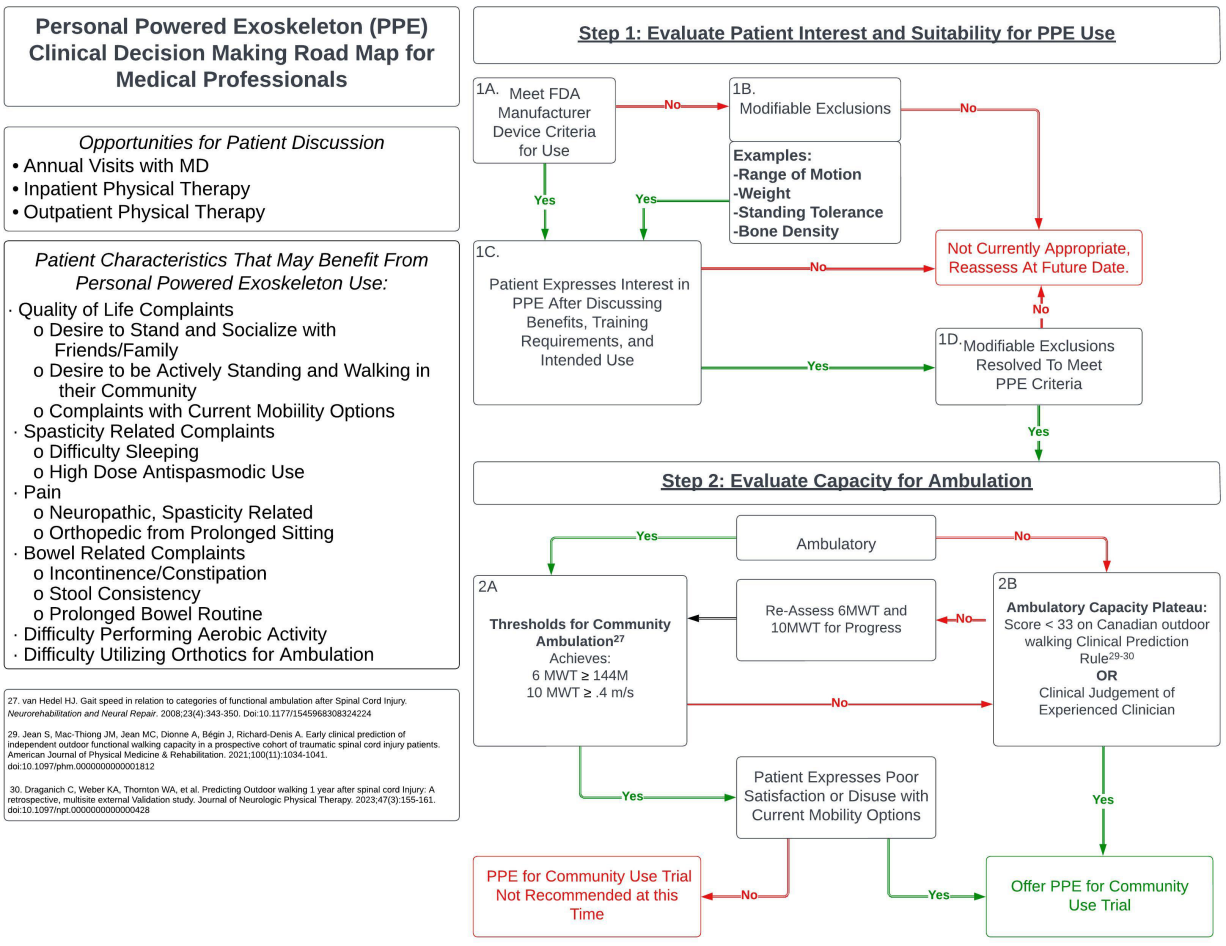
Algorithm for a powered exoskeleton trial.

There are many factors to consider when determining the appropriate time to offer a trial of a PPE for community use to patients living with SCI. This technology is intended to replace functional ambulation capabilities; therefore, in addition to the inclusion and exclusion criteria for use, clinicians should use their professional judgment in consideration of the individual's current level of ambulation, potential for recovery of functional ambulation, and level of motivation to remain ambulatory. It is recommended that consideration of a PPE for community use occur after the rehabilitation team determines the patient has reached a neurological plateau of ambulatory recovery. As discussed above, exoskeleton technologies that are utilized with a goal to enhance locomotor training and recovery are not considered in this recommendation, although there may be overlap to an individual's exposure to these technologies. The following algorithm (Figure 1) is proposed as a guide for clinicians as they go through the process of determining when to offer a PPE trial with a patient who expresses interest and meets usage criteria.

## 3 Step 1: Evaluate to determine patient interest and suitability for PPE use

### 3.1 Criteria for use

Before discussing a PPE for community use with a patient, the clinician must determine if the individual meets the minimum FDA inclusion and exclusion criteria for PPE use (Table 1) (5, 6). The following table is provided as a general guideline of PPE indications and contraindications. Specific criteria may vary by device, so it is recommended that clinicians work directly with manufacturers or therapists certified in the specific PPE of interest to evaluate patient eligibility.

**Table 1:** FDA approved manufacturer inclusion and exclusion criteria.

FDA Approved manufacturer indications (5, 6)
Injury level T3 to L5 AIS A–D
At least 18 years of age
Height between 61″ (155 cm) and 75″ (191 cm) • Upper Leg Length 14.6″ (35 cm)–19.3″ (48.5 cm) • Lower Leg Length 16.5″ (42 cm)–21.7″ (55 cm) • Hip Width 13.3″ (34 cm)–16.6″ (42.2 cm)
Bodyweight ≤250 lbs (113 kg)
Sufficient upper body strength, range of motion, and coordination
Able to tolerate 30–45 min of standing
Skeleton does not suffer from any fractures
Healthy bone density^a^
DEXA score cut off recommendations • <−3.5 total hip BMD T-score • < 0.60 gm/cm^2^ knee BMD
Appropriate range of motion • Hip Ext ≥0° • Knee Flex ≤10° • Ankle Dorsiflexion ≥0°
^a^FDA inclusion criteria states “healthy bone density”. Cutoff scores of −3.5 total hip BMD T-score and 0.60 gm/cm^2^ Knee BMD have been cited in literature as appropriate thresholds to reduce the risk of fracture based on available evidence (7–10)
FDA approved manufacturer contraindications (5, 6)
History of severe neurological injuries other than SCI (MS, ALS, TBI, CVA)
Severe concurrent medical conditions that interfere with safe device use or walking: • Infections, respiratory issues, severe visual impairment, excessive pain, joint instability, or severe arthritis after total/partial lower limb joint replacement • Myocardial infarction/angina/ischemic heart disease within last 6 months • Uncontrolled diabetes • Unresolved DVT • History of renal dialysis • Orthostatic hypotension or essential hypertension • Uncontrolled blood pressure • Colostomy bag • Scoliotic deformity (III-IV)
Psychiatric/cognitive impairment
Uncorrectable equinovarus foot deformation
Unstable or unhealed fractures of the spine, pelvis, and/or lower extremities
Amputations and lower limb prostheses
Uncorrectable leg length discrepancy >2 cm when using additional correction tools
Severe spasticity • Modified ashworth >3

Table references are inclusive of all currently available PPE. Consult device manufacturer for use criteria specific to that PPE.

### 3.2 Modifiable exclusions

If the patient does not currently meet use criteria due to modifiable exclusions such as range of motion, weight, standing tolerance, or bone density, the clinician should inform that individual why they are not currently appropriate, what steps are necessary to meet specific PPE criteria, and discuss if they are motivated to correct the issue(s). If the patient expresses the desire to correct these issue in order to move forward, appropriate interventions should be recommended to best address the exclusion(s).

### 3.3 Training requirements, intended use, and benefits

Once eligibility has been determined based on use criteria, including modifiable exclusions that can be resolved, the clinician should assess patient interest in a PPE for community by discussing the following topics:

#### 3.3.1 Training requirements

While a PPE provides the means to ambulate with a trained companion with less effort and faster speeds than traditional orthotics, it will not be effortless. A patient cannot take their PPE for home use until completion of the manufacturer's FDA approved skills checklists with a trained therapist has been achieved for both the patient and their companion. A companion is any family member or friend willing to take part in training by attending several of the patients' therapy sessions to be certified on how to support them during PPE use. The companion must always be present with the patient when utilizing their PPE as part of FDA guidelines once training is complete. Acquiring the proficiency necessary to complete the manufacturer skills checklists will require multiple sessions with a therapist certified in that specific PPE. Length of training will vary by patient and the specific PPE they are using, with one manufacturer identifying an average of 30–40 sessions to complete the necessary skills for community use. While PPE ambulation is considered by some patients to be a moderate intensity exercise that places a varying level of demand on the upper extremities, depending on factors such as device proficiency and level of injury, training effort will decrease as the patient progresses improving ease of use.

#### 3.3.2 Intended use

Establishing realistic expectations for use with the patient is paramount in making an informed decision. They must understand that a PPE will function as an adjunct to their wheelchair, allowing ambulation in the community and engagement in activities from a standing position, but will not replace their wheelchair for primary mobility. A PPE for community use can provide as much as 100% support to the patient for standing and limb advancement; however, the use of an assistive device for balance support is required. Appropriate terrains are those with firm, level surfaces such as roads, sidewalks, and indoor areas. As previously stated, a trained companion must always be present to supervise and support the patient during activities. As with all physical activity, frequency and duration play an important role in maximizing any potential physiological benefits. While no current recommendations exist regarding a minimum amount of PPE use required to achieve literature-reported benefits, it should be emphasized that there is limited potential without consistently engaging in PPE ambulation. Transporting a PPE in the community varies based on device capability and should be discussed with the specific device manufacturer to determine the recommended transport method. For certain PPE's, the ability to navigate stairs and curbs has been recently approved by the FDA for use in the United States (11), offering patients access to places that may have been previously unavailable due to wheelchair access limitations.

#### 3.3.3 Early evidence of benefits from PPE use

##### 3.3.3.1 Cardiorespiratory function

For individuals living with SCI whose primary mobility is wheelchair use, prolonged sitting is unavoidable. To help combat potential comorbidities, the WHO Guidelines on Physical Activity and Sedentary Behavior recommend that individuals living with disability should perform at least 150–300 min of moderate intensity aerobic activity weekly for substantial health benefits (12). For individuals with SCI, generating a cardiovascular response primarily involves activities from a seated position focusing on upper extremity use. With activities of daily living (ADL) also requiring significant upper extremity involvement, shoulder pain and possible injury from overuse is a common issue (13). Alternative aerobic activities, such as functional electrical stimulation cycling, use electrical impulses to contract the lower extremities in conjunction with a pedaling motion, but require patients to be peripherally innervated to achieve a contraction. RGO's, HKAFO's, or KAFO's allow some individuals with paraplegia to ambulate, but require significant exertional demands with decreased gait speeds (3) impacting both the duration and distance an individual can ambulate for aerobic benefit. The early evidence of PPE ambulation's positive effects on cardiorespiratory function offers a promising alternative with demonstrated improvements in cardiorespiratory function, such as Oxygen Consumption (VO_2_), Cost of Transport (CT), Forced Vital Capacity, and Forced Expiratory Volume in 1 s (FEV_1_) (14–16). 60 sessions of exoskeleton walking over 20 weeks of training was shown to improve participant VO_2_ by an average of 3 ml/kg/min and reduce CT by an average of 2.79 ml/kg/m in individuals with SCI between T1 and T11 (15).

##### 3.3.3.2 Bowel function

Bowel function in the SCI population is often a multi-faceted approach to management that can require a significant amount of time and effort. Adriaansen et al. (2015) evaluated outcome measures from 258 individuals with SCI regarding management of neurogenic bowel in individuals at least 10 years post-SCI. They found that 74% used ≥ one conservative management technique, 45% reported perianal problems, 36% reported severe neurogenic bowel disorder, and 34% reported an average defecation time greater than 30 min (17). Decreased bowel program time (17–19), normalized stool consistency (17, 19), and lessening incontinence and constipation (18) have been reported as positive impacts from exoskeleton use, as well as improved evacuation frequency, decreased laxative/stool softener use, and stool consistency (20).

##### 3.3.3.3 Musculoskeletal and/or neuropathic pain and spasticity

Spasticity can impact multiple aspects of life for individuals with SCI including sleep, comfort, mobility, and ADL's (21). In a 2022 survey of 1076 individuals with SCI, the five most common problematic experiences among patients who reported negative effects of spasticity were all-day stiffness, interference with sleep, painful spasms, perceived link between spasticity and pain, and intensification of pain before spasms with respondents indicating that stretching (48%) and exercise (45%) improved spasticity more than antispasmodic medications (38%) (22). While research on the impact of exoskeleton ambulation on spasticity and pain is limited, studies have indicated a positive effect on perceived spasticity (19, 23, 24) and Modified Ashworth Scale scores (19, 23) after powered exoskeleton use, as well as a reduction in pain for those individuals whom pain was reported (23, 24). This lends significance to the impact of exoskeleton ambulation on spasticity and pain through its ability to provide aerobic exercise while moving a patient's lower extremities through a range of motion that can provide passive stretching.

##### 3.3.3.4 Quality of life

Common issues associated with a lower quality of life (QoL) in individuals with SCI include neuropathic pain, spasticity, musculoskeletal pain, pressure injuries, and constipation (25). While these issues are of undoubted importance for the long-term health of individuals with SCI, the ability to interact with peers and the community is an aspect of QoL that also plays an important role. After 2 months of exoskeleton training Individuals with chronic SCI reported improved social functioning, mental health, and general health perception subdomains on the SF-36ww (26). A 2016 case study of a 22-year-old male at one year post injury also found that, after 6 months of PPE training, improvements were found in six out of eight thematic areas of the SF-36 with the patient capable of ambulating independently with supervision of his companion, supporting the positive impact of community exoskeleton use on QoL (27). While these studies had limited participants, the evidence suggests the potential for PPE use to positively impact QoL. The authors suggest that this improvement in social functioning is potentially related not only to the experience of training in a powered exoskeleton but the opportunity to interact at eye level (26).

### 3.4 Modifiable exclusions resolved

Once the patient has addressed and corrected any identified modifiable exclusions upon follow up examination through prescribed interventions, they should then progress to Step 2 to evaluate their capacity for ambulation.

## 4 Step 2: Evaluate capacity for ambulation

### 4.1 Thresholds for community ambulation

For individuals who are ambulatory with traditional orthotics, we propose using the following values based on van Hedal et al. (2009) for determining Functional Limited Community Ambulation with traditional orthotics (28).

• Achieve ≥144 M on the 6 min Walk Test (6MWT)
• Achieve ≥.4 m/s on the 10 m Walk Test (10MWT)

Previous studies have utilized a cutoff speed of.17 m/s when establishing enrollment criteria for powered exoskeleton use (29), however, this threshold falls below established cut-off speeds necessary for limited community ambulation and may exclude appropriate candidates. If an individual is unable to achieve these minimums with traditional orthotics, then an exoskeleton trial may be appropriate. Patient satisfaction with their orthotics also plays a significant role in their continued utilization; if they attains these minimum values but expresses that they are unlikely to remain ambulatory at that level using their current orthotic, an exoskeleton trial should still be considered.

### 4.2 Ambulatory capacity plateau

While individuals with motor complete or incomplete injuries can be appropriate candidates for PPE use, it can be difficult for clinicians to determine a patients' capacity to achieve community ambulation. To aid clinicians in their decision-making regarding rehabilitation resources and strategies, the Canadian outdoor walking Clinical Prediction Rule (CPR) was proposed to help clinicians predict the percent probability of return to independent outdoor functional walking capacity at 1 year post traumatic SCI (30). Further validated in 2023 by Draganich et al., researchers found that “A CPR of 33 or more was identified as the optimal predictive CPR threshold to predict outdoor walking 1 year after SCI” (31). With its high cross-validated accuracy, we recommend using this CPR to identify individuals with limited potential to achieve outdoor ambulation at 1 year post injury. For individuals with motor incomplete injuries, locomotor training has been demonstrated to yield improvements in walking ability within the 1st year post injury and beyond, with more significant changes in walking measures occurring the closer to initial injury the locomotor training occurred (32, 33). As such, it is recommended that clinicians use their clinical experience and judgement in combination with the CPR, patient exposure to locomotor training, and objective outcome measures to assess the patients capacity to improve their ambulatory ability to community ambulator. If determined by clinician judgement that the patient has not yet achieved a plateau in their capacity to ambulate, it is encouraged that locomotor training be provided with periodic re-assessment of the 6MWT and 10MWT until a sufficient lack of objective and subjective improvement has occurred for the clinician to determine a plateau in ambulatory capacity.

## 5 Limitations

While current literature continues to point towards positive benefits from PPE use, it is important to note that due to the limited population of appropriate candidates for study, sample sizes are cited in the research as a limiting factor. Heterogeneity among study protocols and SCI participants, as well as a lack of long-term studies on the impact of exoskeleton use, have been cited as focus areas for further research. Narrower ranges of injury severity and neurological injury levels are recommended to help strengthen findings regarding impact on secondary complications (34). In a 2021 article by Kandilakis and Sasso-Lance entitled “Exoskeletons for Personal Use After Spinal Cord Injury,” the authors express optimism about the potential for powered exoskeletons to be successfully used in the home and community. They also highlight the need for further research regarding the impact of home/community use on participation and QoL, as well as ways to reduce comorbidities and improve overall health (35). The FDA requirement that a trained companion be present during use, prohibitive financial costs, and lack of medical insurance coverage have also been cited as barriers to accessibility of the technology (36). However, with the recent rule finalization by the Center for Medicare and Medicaid Services to establish PPE within the brace category for eligible beneficiaries (37), there exists now a coverage pathway for eligible individuals outside of the VHA (38).

## 6 Summary

Although opportunities exist to expand the breadth of research regarding PPE use, the current literature points towards a demonstrated positive effect from PPE use in the appropriate population of individuals living with SCI. PPE's provide individuals living with SCI who cannot functionally ambulate the ability to participate in activities inside and outside of their home for aerobic exercise, reducing secondary health conditions, and improving quality of life by engaging in social activities with peers from a standing position. The algorithm presented here is intended to help clinicians make decisions about when and how to educate their patients regarding the potential for PPE use, as well as provide insight into considerations for a PPE trial. For individuals who meet the appropriate criteria, demonstrate the motivation to implement this technology into their lives, and understand the limitations of the devices, a PPE has the potential to be profoundly impactful.

## Data Availability

The original contributions presented in the study are included in the article/Supplementary Material, further inquiries can be directed to the corresponding authors.
